# Minimally Invasive Transforaminal Lumbar Interbody Fusion: A Perspective on Current Evidence and Clinical Knowledge

**DOI:** 10.1155/2012/657342

**Published:** 2012-08-05

**Authors:** Ali Habib, Zachary A. Smith, Cort D. Lawton, Richard G. Fessler

**Affiliations:** Department of Neurological Surgery, Northwestern University, Suite 2210, 676 N. St. Clair Street, Chicago, IL 60611, USA

## Abstract

This paper reviews the current published data regarding open transforaminal lumbar interbody fusion (TLIF) in relation to minimally invasive transforaminal lumbar interbody fusion (MI-TLIF). *Introduction*. MI-TLIF, a modern method for lumbar interbody arthrodesis, has allowed for a minimally invasive method to treat degenerative spinal pathologies. Currently, there is limited literature that compares TLIF directly to MI-TLIF. Thus, we seek to discuss the current literature on these techniques. *Methods*. Using a PubMed search, we reviewed recent publications of open and MI-TLIF, dating from 2002 to 2012. We discussed these studies and their findings in this paper, focusing on patient-reported outcomes as well as complications. *Results*. Data found in 14 articles of the literature was analyzed. Using these reports, we found mean follow-up was 20 months. The mean patient study size was 52. Seven of the articles directly compared outcomes of open TLIF with MI-TLIF, such as mean duration of surgery, length of post-operative stay, blood loss, and complications. *Conclusion*. Although high-class data comparing these two techniques is lacking, the current evidence supports MI-TLIF with outcomes comparable to that of the traditional, open technique. Further prospective, randomized studies will help to further our understanding of this minimally invasive technique.

## 1. Introduction

The advent of minimally invasive surgery has provided surgeons new techniques for treating clinical disease. Within the field of spinal surgery, techniques in lumbar interbody arthrodesis have shown a continued evolution of procedural approach and instrumentation. Minimally invasive spine surgery aims to reduce approach related morbidity, while producing clinical outcomes comparable to its open predecessors. One important example of this is the development of minimally invasive techniques for lumbar interbody fusion, including transforaminal lumbar interbody fusion (TLIF) [1]. 

The MI-TLIF technique, has displayed comparable outcomes to open TLIF, while adding the benefits of less approach-related morbidity, decreased intraoperative blood loss, and shorter hospital stays [2]. However, critics of the technique have noted that the MI-TLIF has longer operative times and exposes patients to increased fluoroscopic radiation. Over the past decade MI-TLIF has been shown to have a number of benefits, especially with regard to peri-operative outcomes. However, it may have its own unique challenges and potential morbidity. Ultimately, comparing the known literature of a traditional, open TLIF approach to published reports on MI-TLIF will identify the unique risks and benefits associated with each. This understanding may help guide improved clinical decision making for patients presenting with lumbar degenerative disk disease. 

In this paper, we evaluate the literature to examine the efficacy of MI-TLIF compared to its open counterpart. In addition, key studies discussing the risks and benefits of MI-TLIF were included to more thoroughly explore the nature of the technique and its application.

## 2. Materials and Methods

In this paper, the authors have used the PubMED/MEDLINE search engines to search for relevant reports addressing the topic of transforaminal lumbar interbody fusion. This was primarily done from Janurary 2000 to Janurary 2012. However, a few historical reports have been added for completeness. Included in this search was the following key phrases: “Minimally invasive,” “transforaminal,” “interbody fusion,” and “lumbar.” We included only English language reports. Further, although articles were first identified by abstract, only full text manuscripts were used to compile this review of the topic. We did not include individual case reports unless associated case series data was included. Further, inclusion criteria were based on the study's contribution in terms of original data, technical variations, and contrasts between open and minimally invasive versions of the procedure ideally completed at the same institution.

In total, 14 articles were selected on the aforementioned basis. All contributed to the established body of the literature pertaining to lumbar arthrodesis techniques, particularly different variants of TLIF. Six of the 14 articles were prospective studies, while the remaining 8 were retrospective (Table 1).

## 3. MI-TLIF Technique

After failed conservative management for a minimum of 6 months, surgery becomes the next therapeutic option for patients presenting with degenerative disc disease (DDD), radiculopathy with spinal instability, and/or grade 1 spondylolisthesis. Initially patients are assessed through radiological investigations including X-ray (AP, lateral, flexion, and extention), and noncontrast lumbosacral MRI. Length of hospitalization is determined by postoperative pain control and functional dependence, with patients of advanced age or medical comorbidities often requiring longer postoperative recovery. However, a majority of patients are admitted the day of surgery and discharged within 24–72 hours after operation.

Under general anesthesia, patients are fixed in a Wilson frame in a prone position. The patient is prepped and draped in standard fashion, and a fluoroscopic C-arm is positioned in the sterile field. Under fluoroscopic guidance the appropriate level is marked and a 3 cm incision is made 4.5 cm of off midline. A k-wire is targeted to the bony complex at the surgical level and serial dilators are consecutively passed to split the muscle fibers. Proper orientation is confirmed by fluoroscopic imaging. A working channel is placed, the dilators are removed, and the channel is secured appropriately for adequate visualization of the medial portion of the facet and inferior lamina. A curette is used to detach the ligamentum flavum from the inferior edge of the lamina, and a kerrison is used to perform the hemilaminectomy. The unilateral facet can be removed using an osteotome or high-speed drill. Following adequate exposure of the disc space, a discectomy is performed using a pituitary rongeur and curette.

**Figure 1:**
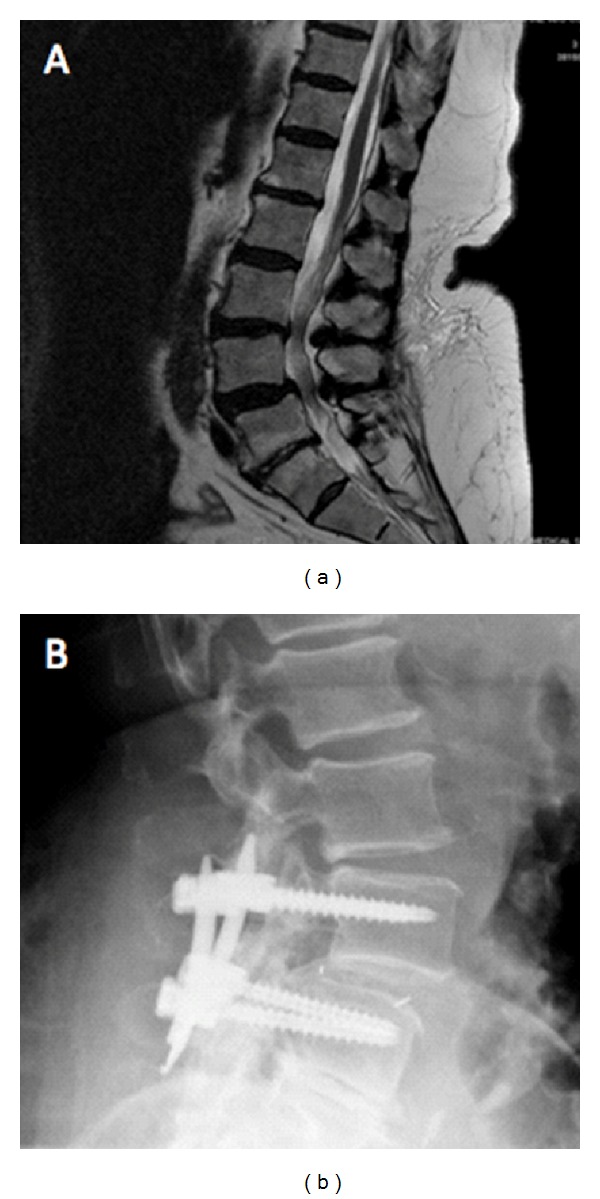
(a) Preoperative lateral MR image of a 72 y/o female patient with back and left leg pain and L4/L5 spondylolisthesis; (b) post-operative lateral MR image from a patient who underwent an MI-TLIF for spondylolisthesis at L4/L5.

**Figure 2:**
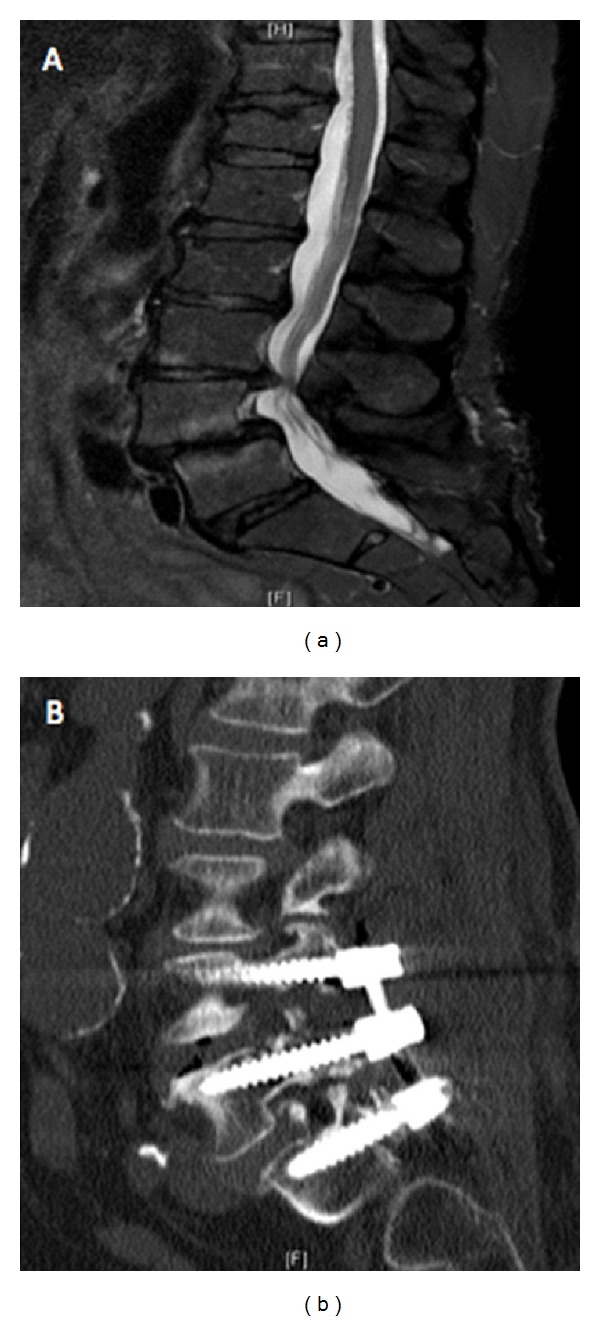
(a) Preoperative lateral MR image of a 66 y/o female with L4/L5 and L5/S1 spondylolisthesis and neuroforaminal stenosis; (b) Post-operative lateral MR image from a patient who underwent an MI-TLIF for spondylolisthesis at L4/L5, L5/S1.

Curved and angled curettes and a disc scraper are then used to prepare the end plate. An appropriately sized interbody spacer is inserted into the disc space, and a half sponge of BMP is packed into the disc space. Fluoroscopy is used to confirm proper positioning of the interbody cage. After removal of the working channel, a jamshidi needle is localized to the unilateral pedicle either above or below the discectomy level, and positioning is checked using fluoroscopic imaging. A K-wire driver is used to insert a guide wire into the superficial portion of the pedicle. A SEXTANT percutaneous screw system (Medtronic Inc; Memphis, TN) is used to pass a cannulated pedicle screw over the K-wire and into the pedicle under fluoroscopic guidance. This is repeated at all desired pedicles on either side. The SEXTANT holding sleeves are mated, the percutaneous rod holder and guide are attached, and a small skin incision is made to pass the rod percutaneously through the screw head. After correct positioning of the rod is confirmed with fluoroscopy, the screw head is tightened, the rod holder is released, and the holding sleeve is removed. Skin closure is accomplished in the standard fashion. For a full detailed description see Lawton et al. [18], see Figures 1 and 2 for illustrative cases from patients treated with the MI-TLIF procedure.

## 4. Review of the Literature

As noted, our review included 14 articles. Follow-up times ranged across all articles from 6 months to 42 months. The mean follow-up was 20 months, with a mean patient cohort of 52 patients. Within seven of the articles that directly compared outcomes of open TLIF with MI-TLIF, mean duration of MI-TLIF surgery was 220 minutes, compared to 218 minutes for its open counterpart. Furthermore, blood loss was found to be on average 282 mL in MI-TLIF cases, while open TLIF resulted in 693 mL of blood loss. The length of stay for MI-TLIF was found to be 5.6 days, while open TLIF had patients in the hospital for an average of 8.1 days (see Table 2). 

### 4.1. Complications

Though the literature displayed possible benefit of MI-TLIF relative to its open counterpart, both procedures are associated with possible complications. Major sources of complications shared by MI-TLIF and Open TLIF are allograft malposition, pedicle screw malposition, and infection [8]. Some minor complications found in both open and MI studies were hematoma, anemia, and cerebrospinal fluid leakage [8]. In both lumbar arthrodesis techniques, placement of a k-wire is necessary, and this k-wire is held in place to formally place the expandable retractor moving the k-wire could result in entrance to the vertebral canal and possible damage to the nerve roots or cauda equina, which had the potential to occur in either TLIF technique [9]. 

Each approach was also associated with its own unique complications. Complications more likely to be found in the open TLIF approach include infections and muscular trauma as a result of the increased exposure and soft tissue dissection [9]. In addition, increased exposure has been shown to be potentially associated with 23.5% of reported complications being infectious in nature, within the open TLIF studies. Open TLIF may have a slightly lower rate of neurological complications, for neurological deficits were a considerably lower proportion of total complications, 11.8%, when compared to MI-TLIF's 20.7%. However, there were a greater variety of unique complications to open TLIF, as shown by 23.4% of complications coming in the form of dural tears, ileus, and atelectasis among others. Please refer to Table 3 for further analysis. 

In the MI-TLIF literature reviewed, many authors discussed the challenging learning curve associated with MI-TLIF, which makes certain complications, particularly those related to instrumentation more likely [5]. Endoscopic visualization of the spinal structure limits the field of view for the surgeon, making identification of already unfamiliar landmarks even more difficult. Though visualization techniques have improved over time, percutaneous fixation systems do not have the ability to reposition three dimensionally [10]. Tubular dilator retractors can result in poor decompression while resulting in higher rates of neurological injuries [4]. Of all complications presented in the MI-TLIF comparative literature, approximately 1 in 5 were related to neurological complications (Table 4). Schizas et al. wrote of possible inexperience leading to inappropriate placement of transpedicular screws, and inadequate preparation of intervertebral cage and fusion site which can lead to further instrumentation related complications. 

The operative surgeon additionally must be familiar with 3D lumbar anatomy and be able to carefully interpret 2D radiographic images to make a mental reconstruction. This is a unique skill and one that is not as critical with a traditional, open approach. The surgeon must be able to read anterior-posterior and lateral imaging in order to accurately insert percutaneous pedicle screws, thereby allowing for possible misinterpretation leading to complications [14]. Screw misplacement and cage migration or subsidence accounted for 44.8% of complications reported in MI-TLIF comparative studies. 

Radiation exposure is another area of interest. MI-TLIF itself presents with increased risk to the surgeon related to increased radiation exposure due to lengthened intraoperative fluoroscopy times. Though many may claim that a surgeon's experience level with minimally invasive procedures will dictate their fluoroscopy times, some studies found no significant difference as experience increased [7]. Very few studies reported the duration and radiation exposure resulting from X-ray and fluoroscopy. Authors who did report this data found that MI-TLIF had greater duration of radiation exposure for patients undergoing the procedure [3, 5, 7]. Due to the relative recent adoption of MI-TLIF use, the long-term effects of increased radiation exposure have not been evaluated. The development of 2D computer assisted fluoroscopy systems as well as the O-arm is a modern means to decrease this exposure risk. Further, careful attention to radiation safety in the operating room is critical. 

### 4.2. Studies of Note

Following data collection and the literature review, it is clear that there is a paucity of data comparing MI-TLIF and open TLIF. To our knowledge, there remains no high-class studies that directly compare these two techniques. However, smaller studies, both prospective and retrospective in nature, have shown promise in regards to novel MI techniques for TLIF. 

Scheufler et al. compared percutaneous transforaminal lumbar interbody fixation (pTLIF) with mini-open transforaminal lumbar interbody fixation (oTLIF) while utilizing the Wiltse method [10]. They found at 8 month and 16 month follow-up, overall clinical outcome did not differ between the two techniques. However, in terms of pain following the operation, pTLIF resulted in significantly lower levels of pain (*P* < 0.01). Though the study showed no decreased advantages due to the percutaneous approach, a longer prospective study would be needed to further discern the success and functionality of each multilevel fusion. 

In a study examining 42 patients with mean follow-up time of 29 months, Dhall et al. compared mini-open and open TLIF [4]. The authors found that mean estimated blood loss for mini-open (194 mL) was significantly lower (*P* < 0.01) than the open-group (505 mL). The length of stay was decreased for mini-open patients by on average, 2.5 days (*P* < 0.01). However, there were complications of neurologic nature in 2 patients, while 2 other patients required further revision. All 42 patients displayed fusion, and the authors felt that the mini-open technique was a possible substitute to open TLIF.

Schwender et al. performed one of the earlier studies (2001-2002) on 49 patients who had MI-TLIF. Majority of patients in the study either had degenerative disc disease with herniated nucleus pulposus (HNP) or spondylolisthesis [14]. 45 of 49 cases were completed at the L4-L5 or L5-S1 levels. Mean operative times were approximately 240 minutes, approximate blood loss was 140 mL, and hospital stays averaged 1.9 days. Complications were limited to four patients, two of which required screw repositioning while two others developed radiculopathy following the procedure. VAPS changed on average from 7.2 to 2.1 while ODI changed from 46 to 14 from preoperative assessment to final follow-up. Ultimately, all patients in the study had fusion on follow-up imaging. The author believed that MI-TLIF is at least equivalent if not a marked improvement over its open counterpart.

A variation of the accepted microendoscopic discectomy was completed by Isaacs and colleagues, which was termed METLIF [6]. METLIF was completed on 20 patients who had lumbar spondylolisthesis or mechanical back pain. This unique procedure compared favorably to patients who underwent PLIF at the same institutions. METLIF resulted in less blood loss, shorter hospital stays, and decreased postoperative narcotic administration. There were no associated procedural complications associated with the multicenter study. Ultimately, this new variation showed promise. 

Schizas et al. examined their institutional experience executing both MITLIF and open midline transforaminal lumbar interbody fusion [5]. Their 36 patient cohort had isthmic spondylolisthesis or DDD which indicated for TLIF. The study found that length of surgery, postoperative pain, analgesia requirements, and VAS/ODI scores were not significantly different between the MI and open procedures. However, they did find that the MI-TLIF did result in significantly less blood loss and a shorter hospital stay. Complications found in the MI-TLIF group, three pseudorthrosis, may have likely been due to the surgeon's gradual adjustment to the novel instrumentation and visualization techniques associated (Table 5). 

## 5. Discussion

Lumbar arthrodesis is an effective method for treating spinal pathology such as spondylolisthesis, DDD, and spinal instability. As minimally invasive spine procedures have emerged, variants such as minimally invasive discectomy and minimally invasive cervical foraminotomies have allowed for reduced complications related to tissue trauma, while reducing blood loss and shortening recovery time [4, 8, 19, 20]. However, no procedure comes without inherent risks. Due to MI-TLIF being a novel procedure for some surgeons, it takes increasingly longer for them to become effective in carrying it out. Villavicencio et al. compared safety and effectiveness of MI-TLIF and open TLIF, showing similar long-term outcomes over the course of the 37.5-month follow-up period [8]. Assigning 63 patients to the open arm and 76 patients to the minimally invasive arm of the study, the authors matched by prior lumbar surgery, diagnosis, and levels at which fusion was performed. They found significant improvement in mean estimated blood loss (*P* < .0001) for MI (163.0 mL) versus the open TLIF (366.8 mL). The study found improvements (*P* = 0.02) in mean duration of hospitalization in MI-TLIF (3 days) relative to their open counterparts (4.2 days). In addition, rates of neurological deficit were significantly higher (*P* = 0.02) in the minimally invasive arm of the study (10.2%) compared to the open cohort (1.6%). Operative times, mean change in VAS scores, patient satisfaction, all significantly favored the open TLIF procedure. The authors hypothesized that the neurological deficits and other factors in favor of open TLIF could have occurred as a result of the surgical learning curve. 

Once the procedure is mastered, its application can positively impact patient care in numerous ways. But, the fundamental advantage of MI-TLIF comes from its decrease in tissue trauma and overall exposure of the patient. This can reduce infection, blood loss, and time to recovery. A prospective cohort study was carried out by Shunwu et al. with 62 patients that had undergone single level TLIF by a single surgeon in a single hospital [9]. One cohort of 32 patients underwent MI-TLIF with the tubular retractor system, while the remaining patients underwent open TLIF. Serum creatine kinase levels, a measure of soft-tissue trauma, was measured on the third postoperative day. Also, time to ambulation and number of transfusions were also measured in the study. Shunwu and colleagues found that MI-TLIF resulted in significantly lower serum creatine kinase levels were found, while patients needed less transfusions and were able to walk earlier than their open counterparts. When comparing the two approaches, this study displayed that MI-TLIF still proposes significant quantifiable benefit in terms of decreased soft tissue trauma. 

As a minimally invasive procedure, MI-TLIF can be utilized to treat particular pathologies, while maintaining the same high levels of clinical success as the open TLIF, even with over two years of follow-up. Thus, the long-term results are comparable to that of open TLIF. Park and Foley contributed an article to the literature that described MI-TLIF in 40 consecutive patients who were diagnosed with spondylolisthesis [16]. Their percutaneous approach resulted in reduction of spondylolisthesis in all cases, with an average follow-up time of 35 months. The average ODI decreased from 55 to 16, while the VAS scores decreased from 65 to 8 in leg and 52 to 15 in back. The average reduction in forward translation was 76%. This was yet another proof of MI-TLIF being a possible replacement to open TLIF in patients with degenerative or isthmic spondylolisthesis. In a prospective study that contrasted clinical and imaging outcomes for MI-TLIF and open TLIF procedures, Peng et al. found that MI-TLIF had equivalent long-term outcomes with open TLIF [3]. The patient cohort had 29 patients in each arm of the study, and 48 of 58 patients were women. The study examined, fluoroscopic times and found that MI-TLIF had significantly (*P* < 0.05) longer (105.5 seconds) compared to open (35.2 seconds). Thus, it is clear that the MI-TLIF cases ran significantly longer overall. Then, the authors discussed the significantly less blood loss, less morphine, and short hospitalization utilized for patients in the MI-TLIF cohort. Yet, open TLIF and MI-TLIF both were very similar in providing significant benefit to patients when rated by ODI, NASS, and VAS, all at follow-ups of six months and two years. In addition, there was no significant difference between open and MI-TLIF in terms of fusion rates, both which were approximately 80%. Peng and colleagues presented data that was supportive of MI-TLIF in terms of pain, hospitalization, and recovery, while at the same time retaining the high-fusion rate associated with open TLIF at two year follow-up. 

Aside from particular pathologies that would benefit from MI-TLIF, there are certain populations that could benefit from the decreased tissue disruption and decreased blood loss. In elderly patients, Lee et al. completed a retrospective review of 27 consecutive cases and found a low complication rate and beneficial outcomes for patients over the age of 65 [12]. The average age of patients in the study was approximately 70 years, and each underwent a mini-open TLIF. They were then followed up for three years, displaying fusion rates of nearly 80%, similar to that seen in other studies. However, 44% of patients displayed adjacent segment degeneration, which was statistically significant in terms of its relation to sacral tilt following the procedure (*P* = 0.006). Two patients experienced minor complications in the perioperative period, one being a drug eruption and the other a urinary tract infection. Overall, the authors strongly felt that mini-open TLIF is a low-risk, beneficial option for the elderly.

## 6. Conclusion

Though the studies presented displayed heterogeneous patient populations with different indications for lumbar arthrodesis, there were many patterns seen across studies. Aside from possible complications such as screw displacement and neurological deficit, which were often related to a steep learning curve, MI-TLIF displayed no significant disadvantages when compared to open TLIF or other standard lumbar fusion techniques. The risks of blood loss, narcotic administration, pseudorthrosis, and infection all are equivalent if not decreased when utilizing MI-TLIF as a possible technique. Various postoperative recovery and pain rating scales often showed consistent improvement across many of the studies presented herein. MI-TLIF and open TLIF are quite similar in absolute indications and often present with similar complications, thus a randomized clinical trial would be beneficial in further elucidating the risks and benefits associated with each. As other variations emerge for MI-TLIF, such as METLIF, there is still need for an overall meta-analysis of all available data, comparing minimally invasive technique to traditional, open procedures.

## Figures and Tables

**Table 1:** Summary of research studies reporting data on MI-TLIF.

Author (year)	Study design	Follow-up	Number of patients	Significant results
Scheufler et al. (2007) [10]	Retrospective	8 months, 16 months	53	OR time equivalent between pTLIF and mini-open TLIF Blood loss and postoperative pain reduced reduced in pTLIF

Villavicencio et al. (2010) [8]	Retrospective	37.5 months	63 and 76 patients	Mean blood loss lower in MI-TLIF Mean duration of hospital stay shorter in MI-TLIF Rate of neurological deficit was greater in the MI-TLIF group

Schizas et al. (2009) [5]	Prospective	22 months (MI) 24 months (O)	36 patients (O = 18, MI = 18)	MI-TLIF: decreased blood loss, shorter hospital stay, and decreased pain Steeper learning curve in MI-TLIF

Dhall et al. (2008) [4]	Retrospective	24 months (MI) 34 months (O)	21 (MI) 21 (O)	MI-TLIF: less blood loss, shorter LOS

Jang and Lee (2005) [13]	Pilot	30 months	100 consecutive patients	Significant reduction in pain, ODI, and TIS Improvement in lordosis from 2^°^ to 9^°^, anterior disc height 6 to 14 mm, and posterior disc height from 4 to 8 mm

Peng et al. (2009) [3]	Prospective	6 months, 2 years	29 (MI), 29 (O)	MI-TLIF: fluoroscopic time increased, longer operative times, less blood loss, decreased morphine use, and decreased LOS

Beringer and Mobasser (2006) [15]	Prospective	6 months	8	All had solid bone fusions

Park and Foley (2008) [16]	Retrospective	Minimum 24 months, Mean 35 months	40	Mean ODI 55→16 post-op Mean leg and back pain VAS 65 and 52, improving to 8 and 15 Reduction of spondylolisthesis was achieved in all cases, with a mean decrease in forward translation of 76%

Deutsch and Musacchio (2006) [11]	Prospective	6–12 months	20	85% had >20 point reduction in ODI ODI 57→25 VAS 8.3→1.4

Jang and Lee (2005) [13]	Prospective	19 months	23	NRS back pain 7.5→2.3 NRS leg pain 7.4→0.7 Mean ODI 33.1→7.6

Isaacs et al. (2005) [6]	Retrospective	n/a	20	METLIF: less blood loss, less postoperative wound drainage, no dural violation, less pain medication, and shorter LOS

Shunwu et al. (2010) [9]	Prospective cohort study	24–42 months	32 (MI), 30 (O)	MI: reduced blood loss, les postoperative back pain, lower serum creatine kinase, shorter time to ambulation, and shorter LOS

Wang et al. (2010) [7]	Prospective	Minimum 13-month follow-up	MI = 42, O = 43	MI: reduced blood bloss, less postoperative back pain, shorter LOS, greater radiation time

Foley et al. (2003) [2]	Retrospective	12–20 months, mean 22 months	39 patients	Twenty-six had excellent outcomes and 12 had good ones, as determined by the modified MacNab criteria

Schwender et al. (2005) [14]	Retrospective	22.6 mean follow-up	49 patients	Estimated blood loss of 140 mL, mean length of hospital stay 1.9 days, and all 45 patients presenting with preoperative radiculopathy had resolution of symptoms

Dong et al. (2008) [12]	Retrospective	38.6 mean follow-up	27 patients	Solid fusion in 77.8% of patients, clinical success achieved in 88.9% of cases

Anand et al. (2006) [17]	Prospective	30	100	Improvement in VAS, ODI, TIS, and NRS for back, 99% fusion

**Table 2:** Comparative studies basic data.

Author	Mean duration of surgery MIS	Mean duration of surgery open	MIS blood loss	Open blood loss	Length of stay MIS	Length of stay open
Villavicencio et al.	222.5	214.9	163 mL	366.8	3	4.2
Shunwu et al.	159.2	142.8	399.8	517	9.3	12.5
Wang et al.	156 (X-ray 84)	145 (37)	264	673	10.6	14.6
Peng et al.	216.4 (fluoro 105.5 s)	170 (35.2)	150	681	4	6.7
Schizas et al.	348 (X-ray 2.7 cGy/cm^2^)	312 (1.8)	456	961	6.1	8.2
Dhall et al.	199	237	194	505	3	5.5
Isaacs et al.	300	276	226	1147	3.4	5.1

**Table 3:** Complications found in studies comparing open TLIF to MI-TLIF.

		Complication type		Complication rate	
Author	Year	Open	MI	Open	MI
Peng et al. [3]	2009	Atelectasis-(1) UTI-(2) Infection-(1)	Infection-(1)	13.5%	6.9%

Dhall et al. [4]	2008	Radiculitis (1) Misplaced screw-(1)	Transient L-5 sensory loss (2) Misplaced screw (1) Cage migration (1)	2%	5%

Schizas et al. [5]	2009	NR	Increased pseudarthrosis	2%	6%

Isaacs et al. [6]	2005	Infection Fluid shift/blood transfusion complications Positioning-related neuropraxia of the upper extremity	Transient leukopenia (1)	6%	0%

Wang et al. [7]	2010	Pedicle screw malposition (1) Dural tears (2)	Radiculopathy (2) Small dural tear (1)	4%	5%

Villavicencio et al. [8]	2010	CSF leak	Neurological deficit > 3 mos Pedicle screw malposition with reoperation	31.7%	31.6%

Shunwu et al. [9]	2010	Superficial wound infection (1) Deep wound infection (1) Deep venous thrombosis (1) Ileus (1)	Screw malposition (2) Superficial wound infection (1) Ileus (1)	5%	6%

**Table 4:** Complication rate by TLIF approach.

Complications	MI	Open
Infection	6.9%	23.5%
UTI	3.4%	11.8%
Neurologic deficits	20.7%	11.8%
Screw/Cage complications	44.8%	11.8%
CSF leak	10.3%	5.9%
Blood transfusion/coagulation	3.4%	11.8%
Other	10.5%	23.4%

**Table 5:** MI-TLIF complication types and complication rates.

Author	Year	MI-TLIF complication type	MI-TLIF complication rates
Scheuffler et al. [10]	2007	CSF leak (1)	1.9%
Deutsch and Musacchio [11]	2006	Misplaced screw (1) CSF leak (2)	4
Dong et al. [12]	2008	UTI (1) Drug reaction (1) Subsidence	7.4%
Jang and Lee [13]	2005	Subsidence (3) Screw failure (1)	17.4%
Scwender et al. [14]	2005	Misplaced screws (2) Radiculopathy (2)	8.2%
Beringer and Mobasser [15]	2006	NR	NR
Park and Foley [16]	2008	NR	NR
Anand et al. [17]	2006	NR	NR

## Abbreviations

PLIF: Posterior lumbar interbody fusion
ALIF: Anterior lumbar interbody fusion
TLIF: Transforaminal lumbar interbody fusion
MI-TLIF: Minimally invasive transforaminal lumbar interbody fusion
DDD: Degenerative disc disease
LOS: Length of stay
VAS: Visual analog scale
ODI: Oswestry Disability Index.
